# *Paraburkholderia suaedae* sp. nov., a Potential Plant Growth-Promoting Bacterium Isolated from the Halophyte *Suaeda japonica*

**DOI:** 10.3390/microorganisms13112498

**Published:** 2025-10-30

**Authors:** Sunho Park, Hyunji Lee, Subin Yook, Chunghwan Baek, Jisu Kim, Seunghui Kwak, Taeho Na, Taegun Seo

**Affiliations:** Department of Life Science, Dongguk University-Seoul, Goyang 10326, Republic of Korea; eksvnd97@dgu.ac.kr (S.P.); guswl4851@dgu.ac.kr (H.L.); qw745043@gmail.com (S.Y.); qazx12300@naver.com (C.B.); didoo0v0@gmail.com (J.K.); sholly0@naver.com (S.K.); thth0417@dgu.ac.kr (T.N.)

**Keywords:** *Paraburkholderia*, plant growth-promoting bacteria, pan-genome analysis, Kyoto Encyclopedia of Genes and Genomes (KEGG), novel bacterial species

## Abstract

A novel bacterial strain was isolated from the roots of *Suaeda japonica*, a halophytic plant inhabiting tidal zones. Phylogenetic, genomic, and phenotypic analyses identified the isolate as a novel species within the genus *Paraburkholderia*, for which the name *Paraburkholderia suaedae* sp. nov. is proposed. The strain exhibits multiple plant growth-promoting traits, including the production of 1-aminocyclopropane-1-carboxylic acid, indole-3-acetic acid, and siderophore, along with the ability to fix nitrogen and solubilize phosphate. Genomic analysis revealed genes associated with enhanced root surface adhesion and rhizosphere survival, such as those involved in thiamine biosynthesis and transport, and biofilm formation via poly-β-1,6-N-acetyl-D-glucosamine (PGA) synthesis. These features suggest the strain’s potential for persistent colonization and beneficial interaction with host plants. Although its direct impact on plant growth has not yet been experimentally validated, the genetic and biochemical evidence supports its potential application in agriculture. The objective of this study was to conduct a polyphasic taxonomic characterization of a novel strain DGU8^T^ isolated from the roots of the halophyte *Suaeda japonica*, and to assess its potential as a plant growth-promoting agent, particularly its tolerance to drought-related osmotic stress.

## 1. Introduction

Climate change has led to an increase in the frequency and severity of natural disasters, particularly drought, posing significant challenges to global agriculture [1]. As climate change accelerates, crops will be increasingly exposed to water scarcity, making the development of drought-resistant plants a priority [2]. One promising approach to enhance drought tolerance involves inoculating crops with beneficial microbial strains, which can strengthen plant resilience under water-limited conditions [3]. To explore such microbial adaptations, we conducted studies in extreme environments such as intertidal zone ecosystems characterized by fluctuating salinity and periodic inundation. These harsh conditions support only highly adaptable plant species, such as *Suaeda japonica* (Chenopodiaceae) [4]. *Suaeda japonica* is an annual halophyte native to the Yellow Sea region of Republic of Korea and Kyushu, Japan. It thrives in saline-affected tidal flats and typically grows to a height of 20–50 cm [5]. Tidal flats formed by alternating low and high tides are ecologically valuable zones that contribute to nutrient cycling and ecosystem restoration [6,7,8]. *S. japonica* has attracted scientific interest due to its diverse biological activities. Its underground parts exhibit antioxidant and reducing properties, and its extracts have been used to synthesize zinc oxide nanoparticles with photocatalytic and antibacterial effects [9,10]. Additionally, its bioactive compounds have demonstrated anti-obesity effects in murine models [11].

Given this ecological and biochemical significance, our study aimed to isolate novel microbial strains from the rhizosphere of *S. japonica* and evaluate their plant growth-promoting potential. Some genera have been isolated from tidal flat sediments, the natural habitat of the halophyte *S*. *japonica*. However, research targeting the specific microbial communities within this plant’s rhizosphere remains limited. Among the microorganisms adapted to such high-salinity environments, the genera *Marinobacter* and *Wenyingzhuangia* are well-documented [12]. In contrast, there are no existing reports on the isolation of the genus *Paraburkholderia* from the rhizosphere of *S*. *japonica* or, more broadly, from any halophyte in a tidal flat ecosystem.

According to the LPSN database (https://lpsn.dsmz.de/genus/paraburkholderia; accessed on 27 October 2025), Sawana et al. reclassified *Paraburkholderia* as an independent genus distinct from *Burkholderia*, designating *Paraburkholderia graminis* as the type species [13]. Members of this genus are predominantly isolated from soil, plant roots, and root-associated environments [14,15,16,17]. Of the 139 recorded species, 96 have been validly published to date [18]. *Paraburkholderia* species are Gram-negative, straight or slightly curved rod-shaped bacteria that are generally aerobic and motile via one or more polar flagella [19]. Most species possess relatively large genomes ranging from 7.0 to 10.0 Mb, with DNA G + C content between 58.9 and 65.0 mol%. Their major fatty acids include C_16:0_, C_17:0_ cyclo, C_19:0_ cyclo *ω*8*c*, and summed feature 8 (C_18:1_ *ω*7*c* and/or C_18:1_ *ω*6*c*) [19,20], while the dominant polar lipids are phosphatidylethanolamine, phosphatidylglycerol, and diphosphatidylglycerol. Several *Paraburkholderia* strains have demonstrated plant growth-promoting capabilities. For instance, *Paraburkholderia* sp. GD17 and *Paraburkholderia phytofirmans* PsJN^T^ have shown beneficial effects on crops such as Chinese cabbage, wheat, corn, and grapevines [21,22]. Similarly, *Paraburkholderia* sp. Msb3 and *Paraburkholderia tagetis* RG36^T^ have been reported to enhance tomato growth [23,24]. These findings underscore the genus’s long-standing relevance in growth promotion research. The first objective of this study was to isolate and taxonomically characterize a novel bacterial strain from the rhizosphere of the halophyte *S*. *japonica* using a multifaceted genomic, phenotypic, and chemotaxonomic analysis. The second objective was to evaluate the strain’s potential as a plant growth promoter through genomic analysis and in vitro experiments, focusing on its ability to withstand drought-induced osmotic stress.

## 2. Materials and Methods

### 2.1. Isolation of Rhizosphere Bacteria

*S. japonica* samples were collected from a natural colony located at 37°40′28.6″ N, 126°22′35.2″ E on Seongmodo Island, Republic of Korea. Ten individual plants were randomly selected at 30 m intervals within the sampling area. Using sterile scissors, root segments were excised and placed into 50 mL conical tubes. Samples were stored in an ice-filled cooler during the 1 h transport.

Upon arrival, roots were rinsed with sterile distilled water to remove surface debris, gently washed with sterile 0.1% Tween 20 for 30 s, and rinsed twice with sterile distilled water. Surface sterilization was performed using 95% ethanol (Duksan, Ansan-Si, Republic of Korea) for 5 min, followed by 1% sodium hypochlorite (Duksan, Republic of Korea) for 5 min, and two final rinses with sterile distilled water [12].

Sterilized root samples were placed in 10 mL of sterile 0.85% (*w*/*v*) saline solution in 50 mL conical tubes, rotated at 180 rpm for 1 h on a rotary shaker, and vortexed for 10 min. Finally, 100 μL aliquots from 10-fold serial dilutions (10^−1^, 10^−2^, and 10^−3^) were spread onto Marine Agar 2216 (MA; Difco, France) and Reasoner’s 2A agar (R2A; MB cell, Seoul, Republic of Korea), followed by incubation at 30 °C for 4 days.

### 2.2. 16S rRNA Amplification and Phylogenetic Analysis

The novel strain DGU8^T^ was sequenced using the universal bacterial primer set 27F/1492R by Solgent Co., Ltd. (Daejeon, Republic of Korea). Sequence assembly and analysis were performed using SeqMan software (version 5.0; DNASTAR, Inc., Madison, WI, USA; https://www.dnastar.com/software/lasergene/) [25] and Chromas (version 2.6.6; Technelysium Pty., Ltd., South Brisbane, Australia. https://technelysium.com.au/wp/chromas/) following established protocols.

The complete 16S rRNA sequence of DGU8^T^ was submitted to the EzbioCloud database (CJ Bioscience, Inc., Seoul, Republic of Korea; http://ezbiocloud.net; accessed on 8 April 2024) for species identification and comparison with closely related strains [26]. The sequence was also deposited in the NCBI GenBank/EMBL/DDBJ database (www.ncbi.nlm.nih.gov/genbank/; accessed on 20 April 2024) and assigned a GenBank number.

Gene sequences of 19 reference strains were obtained from EzBioCloud for comparative analysis. Phylogenetic trees were constructed using maximum likelihood [27], neighbor-joining [28], and maximum parsimony [29] algorithms in MEGA X software (Molecular Evolution Genetics Laboratory, Pennsylvania State University, Pennsylvania, USA; https://www.megasoftware.net/; accessed on 1 October 2025) [30]. Evolutionary distances were calculated using the Kimura-2-parameter model [31], and bootstrap values were derived from 1000 replicates. *Escherichia coli* X80725^T^ (ATCC11775) was used as the outgroup.

### 2.3. Genome Features and Comparative Analysis

Genomic DNA of the novel strain DGU8^T^ cultured on R2A agar at 30 °C for 4 days, was extracted by Macrogen Co., Ltd. (Seoul, Republic of Korea) using a TruSeq DNA PCR-Free kit (Illumina, Inc., San Diego, CA, USA). Whole-genome shotgun libraries were prepared and sequenced using the Illumina NovaSeq X platform. The draft genome was assembled de novo using the SPAdes genome assembler (version 3.15.0) [32], and its completeness was assessed using BUSCO software (version 5.1.3) [33].

Functional annotation and gene prediction were performed using Prokka (version 1.14.6) [34] and BLAST (version 2.17.0) [35] to identify contigs, coding sequences, and genes for tRNA, rRNA, and tmRNA. Plant growth-promoting genes including those involved in phosphate solubilization, nitrogen fixation, pyrroloquinoline quinone (PQQ) biosynthesis, indole-3-acetic acid production, and siderophore synthesis [36,37,38,39,40,41,42,43,44] were identified by comparing Prokka results with the Evolutionary Genealogy of Genes: Non-supervised Orthologous Groups (EggNOG) database (version 4.5) [45].

Genome completeness and contamination were further evaluated using CheckM2 (version 1.1.0; https://github.com/chklovski/CheckM2) [46]. The annotated genome was submitted to the NCBI Prokaryotic Genome Annotation Pipeline (PGAP) (version 6.5; www.ncbi.nlm.nih.gov/genome/annotation_prok) to obtain an accession number [35].

Phylogenetic classification of the novel strain was performed by comparing its genome with those of 96 validly published species of the genus *Paraburkholderia* registered in the NCBI database. A phylogenetic tree was constructed using the Up-to-date Bacterial Core Genes 2 (UBCG2; version 2.0; http://leb.snu.ac.kr/ubcg2), which includes 3508 species and 81 single-copy bacterial core genes [47]. The resulting 97 *Paraburkholderia* species were further analyzed using average amino acid identity (AAI) via the ezAAI tool in EzbioCloud (https://endixk.github.io/ezaai/; accessed on 21 June 2024) [48].

Protein-coding genes were annotated using the Rapid Annotation using Subsystem Technology (RAST) server (https://rast.nmpdr.org/; accessed on 24 June 2024) [49,50,51]. Secondary metabolite biosynthetic gene clusters were predicted using antiSMASH (version 7.0; https://antismash.secondarymetabolites.org/) [52].

Taxonomic relatedness to closely related strains was assessed using average nucleotide identity (ANI) and digital DNA–DNA hybridization (dDDH). ANI was calculated using the Orthologous Average Nucleotide Identity Tool (OAT; https://www.ezbiocloud.net/tools/orthoani; accessed on 24 August 2024) [53], and dDDH values were obtained using the Genome-to-Genome Distance Calculator (GGDC) (version 3.0; http://ggdc.dsmz.de/ggdc.php#) [54].

Genomes of DGU8^T^ and reference strains that clustered within the same clade (based on UBCG analysis) were visualized using the Anvi’o pan-genome workflow (version 8.0). Pan-genome analysis was performed using the presence–absence algorithm (D: Euclidean; L: Ward), identifying singletons, core genes, and single-copy gene clusters (SCGs) [55,56]. Kyoto Encyclopedia of Genes and Genomes (KEGG) orthologs (KOs) were assigned using KofamScan (https://github.com/takaram/kofam_scan; accessed on 1 September 2024) [57], and KEGG Decoder (version 1.3.0; https://github.com/bjtully/BioData/tree/master/KEGGDecoder) was used to map KOs to metabolic pathways and generate heatmaps. KEGG annotations with E-values exceeding 1 × 10^−5^ were excluded from the analysis [58].

### 2.4. Morphology and Chemotaxonomic Characteristics

Strain DGU8^T^ was cultured at 30 °C for 4 days in R2A, tryptone soy agar (TSA; Difco, Eybens, France), Luria–Bertani agar (LB agar; Difco, Eybens, France), nutrient agar (NA; Difco, Eybens, France), and MA to determine the optimal growth medium. For phenotypic comparison, strain DGU8^T^ and 5 reference strains were each inoculated onto optimum agar and cultured at 30 °C for 3 days. Subsequent experiments were conducted in R2A broth (MB cell; Republic of Korea), incubated at 30 °C for 4 days with shaking at 180 rpm.

Temperature tolerance was assessed by incubating the strain on R2A agar plate and incubated for 4 days at various temperatures (at 10, 15, 20, 25, 30, 35, 37, and 40 °C) to determine its temperature growth range. NaCl tolerance was evaluated by culturing in R2A broth supplemented with 0–3.0% (*w*/*v*) NaCl at 1.0% intervals. pH tolerance was assessed by incubating in R2A broth adjusted to pH values ranging from 3.0 to 12.0, in 1.0-unit increments, for 4 days. Buffers used included citric acid (pH 3.0–5.0), sodium dihydrogen phosphate (pH 5.0), phosphate buffer (pH 6.0–8.0), Tris(hydroxymethyl)aminomethane (pH 9.0–10.0), and Na_2_HPO_4_–NaOH (pH 11.0–12.0), each at a final concentration of 50 mM [59].

Drought resistance was assessed by culturing strain DGU8^T^ in R2A broth supplemented with various concentrations of polyethylene glycol (PEG) 6000 (0, 1, 5, 10, 15, and 20%, *w*/*v*; Steinheim, Germany). A 20 µL aliquot of a pre-culture was inoculated into 2 mL of each medium and incubated at 30 °C for 3 days. Bacterial growth was determined by measuring the optical density at 600 nm (OD_600_) using a spectrophotometer (Multiskan GO, Thermo Fisher Scientific Inc., Waltham, MA, USA) and compared to a negative control.

Cell growth was monitored by measuring the absorbance at 600 nm using spectrophotometer (Multiskan GO, Thermo Fisher Scientific Inc., Waltham, MA, USA). Physiological and enzymatic characteristics were analyzed using the API 20NE kit (BioMérieux, Marcy-l’Étoile, France) according to the manufacturer’s instructions. Anaerobic growth was assessed by incubating R2A agar plates in BBL GasPak anaerobic culture vessels at 30 °C for 14 days.

Hydrolytic enzyme activity was assessed by supplementing R2A agar plates with 1% of the following substrates: casein (1% skim milk; BioPure, USA), chitin (1%; Tokyo Chemical Industry Co., Ltd., Tokyo, Japan), carboxymethyl-cellulose (CM-cellulose; 1%; Duksan Co., Gyeonggi-do, Republic of Korea), starch (1%; Sigma-Aldrich, Merck KGaA, Darmstadt, Germany), Tween 20 (1%; BioPure, Woodinville, WA, USA), and Tween 80 (1%; BioPure, Woodinville, WA, USA). Plates were incubated at 30 °C for 7 days, and enzyme activity was evaluated as previously described [60].

Cells cultured on R2A agar for 4 days were stained with 1% (*w*/*v*) phosphotungstic acid and examined using transmission electron microscopy (TEM) to observe morphology and motility. Oxidase activity was determined by the appearance of a purple coloration upon application of 1% tetramethyl-p-phenylenediamine (BioMérieux, Marcy-l’Étoile, France). Catalase activity was confirmed based on bubble formation following the addition of a 3% hydrogen peroxide (H_2_O_2_) solution. Gram staining was performed according to a previously described method [61].

For fatty acid analysis, cells were harvested from R2A agar plates after 7 days of incubation at 30 °C. Fatty acid methyl esters (FAMEs) were extracted using the MIDI Sherlock Microbial Identification System (MIS; version 6.01, database TSBA6; MIDI Inc., Newark, DE, USA) following saponification, methylation, and solvent extraction as described by Kuykendall et al. [62]. Fatty acids accounting for ≥10.0% of the total content were classified as major, while those present at <1.0% were designated as trace (TR). Quinones were extracted using a chloroform/methanol mixture (2:1, *v*/*v*) and analyzed using high-performance liquid chromatography following the method of Minnikin et al. [63].

### 2.5. Functional Traits Related to Plant Growth Promotion

To assess 1-aminocyclopropane-1-carboxylic acid (ACC) deaminase activity, a pre-culture of strain DGU8^T^ was inoculated into Dworkin–Foster (DF) minimal salt medium supplemented with 0.3 mM ACC [64] and incubated at 30 °C for 4 days. ACC deaminase catalyzes the degradation of ACC, a precursor of ethylene, and its activity was confirmed based on growth in the ACC-containing medium.

Nitrogen fixation ability was evaluated using Jensen’s medium, which contained sucrose (20.0 g/L), K_2_HPO_4_ (1.0 g/L), MgSO_4_ (0.5 g/L), CaCO_3_ (2.0 g/L), NaCl (0.5 g/L), Na_2_MoO_4_ (0.005 g/L), FeSO_4_ (0.1 g/L), and agar (15.0 g/L). Nitrogen-fixing ability was indicated by colony formation accompanied by a yellow halo.

Phosphate solubilization was assessed using Pikovskaya (PVK) medium composed of yeast extract (0.5 g/L), dextrose (10.0 g/L), Ca_3_(PO_4_)_2_ (5.0 g/L), (NH_4_)_2_SO_4_ (0.5 g/L), KCl (0.2 g/L), MgSO_4_ (0.1 g/L), MnSO_4_ (0.0001 g/L), FeSO_4_ (0.0001 g/L), and agar (15.0 g/L). The ability to solubilize phosphate was confirmed by the formation of a clear zone around the inoculated strain.

Siderophore production was assessed using universal chrome azurol S (CAS) agar, prepared according to a previously described method [65]. The presence of an orange halo and clear zone around the colonies indicated siderophore production.

Auxin production was assessed using a colorimetric method involving the supernatant of bacterial cultures grown in R2A broth supplemented with varying concentrations of L-Tryptophan (0.5, 0.25, 0.125, 0.0625, 0.0313, and 0%; Sigma-Aldrich, St. Louis, MO, USA) [66]. Cultures were incubated at 30 °C and 180 rpm for 3 days. After centrifugation at 10,000 rpm for 10 min, 1 mL of the supernatant was mixed with 2 mL of Salkowski reagent (98 mL of 35% perchloric acid and 2 mL of 0.5 M FeCl_3_), and the mixture was incubated in the dark at 25 °C for 30 min [67]. The absorbance of the resulting red solution was measured at 530 nm using a spectrophotometer (Multiskan GO, Thermo Fisher Scientific Inc., Waltham, MA, USA), and indole-3-acetic acid (IAA) concentrations were calculated using a standard curve. For each measurement, the absorbance value of a corresponding blank sample (R2A broth with the same L-tryptophan concentration but without bacterial inoculation) was subtracted to correct for any non-biological color development.

Strain DGU8^T^ was inoculated into media formulated for assessing plant growth-promoting traits and incubated at 30 °C for 7 days.

## 3. Results

### 3.1. Isolation of Rhizosphere Bacteria

After 4 days of cultivation, colonies with diverse morphologies and pigmentation were observed. Pure isolation was achieved through repeated subculturing. The isolated strain DGU8^T^ formed circular, convex, ivory-colored colonies and was preserved at −80 °C in 25% (*v*/*v*) glycerol. The strain was designated DGU8^T^ and deposited at the Korean Agricultural Culture Collection (KACC 23737^T^; Department of Agriculture, Agricultural Microbiology Division, Wanju, Republic of Korea) and the Thailand Bioresource Research Center (TBRC 19126^T^; Khlong Nueng, Pathum Thani, Thailand).

### 3.2. 16S rRNA Amplification and Phylogenetic Analysis

The assembled 16S rRNA gene sequence of strain DGU8^T^ registered in EzbioCloud, was 1464 bp in length and classified within the phylum *Pseudomonadota*, class *Betaproteobacteria*, order *Burkholderiales*, family *Burkholderiaceae*, and genus *Paraburkholderia*. Sequence similarity analysis using the EzbioCloud database (version 2025.04.21) revealed the highest similarity with *Paraburkholderia rhynchosiae* WSM3937^T^ (98.35%), followed by *Paraburkholderia panacihumi* DCY115^T^ (98.21%), *Paraburkholderia fynbosensis* WSM4178^T^ (98.14%), *P. panacisoli* DCY113^T^ (98.00%), and *P. phytofirmans* PsJN^T^ (97.94%).

**Figure 1:**
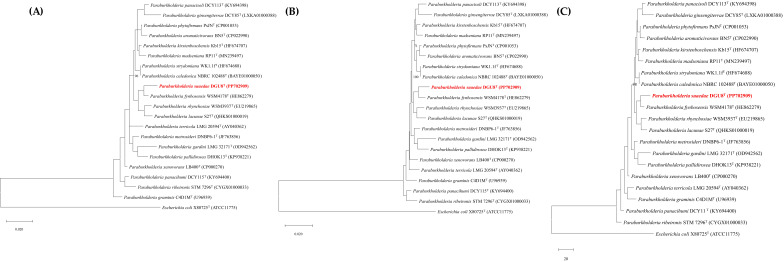
Phylogenetic analysis of the 16S rRNA gene based on (**A**) neighbor-joining (NJ), (**B**) maximum likelihood (ML), and (**C**) maximum parsimony (MP) algorithms. Bootstrap values are shown as percentages from 1000 replicates (above 70%). *Escherichia coli* X80725^T^ (ATCC11775) was used as the outgroup. The sum of branch lengths for the NJ and ML trees was 0.020 (substitutions/site). The tree length for the MP analysis was 20 steps.

Phylogenetic analysis of 19 *Paraburkholderia* species using the NJ, ML, and MP algorithm based on 16S rRNA gene sequences showed that strain DGU8^T^ clustered closely with *P*. *fynbosensis* WSM4178^T^, *P*. *rhynchosiae* WSM3937^T^, and *Paraburkholderia lacunae* S27^T^, confirming its clear inculsion within the genus *Paraburkholderia* (Figure 1). The 16S rRNA gene sequence of strain DGU8^T^ was deposited in the NCBI database under accession number PP702909 (https://www.ncbi.nlm.nih.gov/nuccore/PP702909).

### 3.3. Genome Features and Comparative Analysis

**Figure 2:**
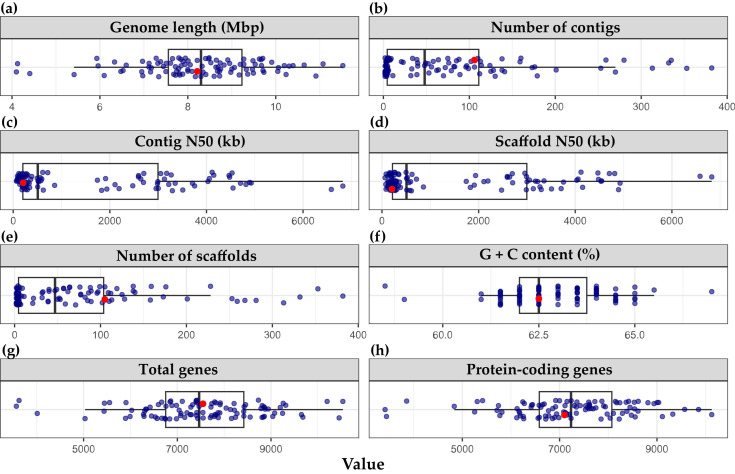
Genome statistics for 97 *Paraburkholderia* species including the novel strain *Paraburkholderia suaedae* DGU8^T^. (**a**) Genome length (Mbp), (**b**) number of contigs, (**c**) Contig N50 (kb), (**d**) Scaffold N50 (bp), (**e**) number of scaffolds, (**f**) G + C content (%), (**g**) total genes, and (**h**) protein-coding genes. The novel strain DGU8^T^ is marked with a red dot.

The draft genome of strain DGU8^T^ was assembled at the scaffold level, yielding a total genome size of 8,214,678 bp. The assembly comprised 105 scaffolds and contigs, with both scaffold N50 and contig N50 values at 207,651 bp, and L50 values of 12 for each. The genome contained 4 rRNAs, 58 tRNAs, 7548 total genes, and 7482 protein-coding genes (https://www.ncbi.nlm.nih.gov/datasets/genome/GCF_040812055.1/; accessed on 22 July 2024). CheckM2 analysis indicated a genome completeness of 100.0% and contamination of 1.13%. Based on the completed draft genome, the G + C content of strain DGU8^T^ was calculated to be 62.5%. Comparative genome statistics of 97 species *Paraburkholderia* using PGAP are presented in Figure 2 and Supplementary Excel S1.

**Figure 3:**
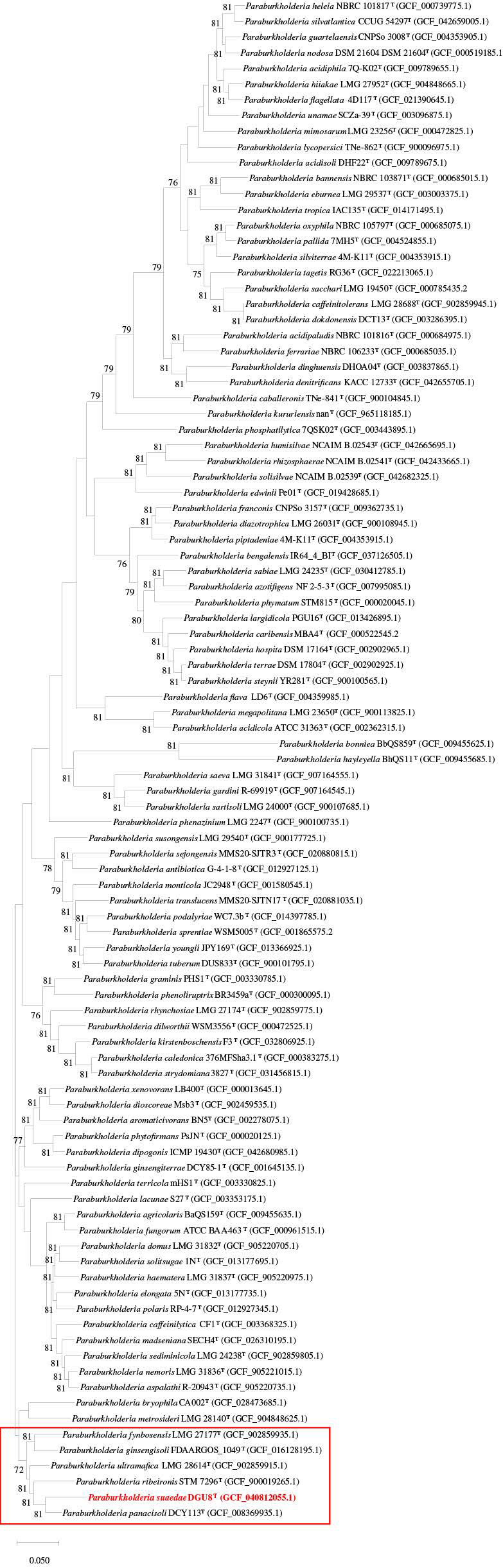
UBCG2 phylogenetic tree constructed from 81 bacterial core gene sequences, illustrating the phylogenetic placement of the novel strain *Paraburkholderia suaedae* DGU8^T^ among 96 *Paraburkholderia* species. Bootstrap values (≥70%) were calculated from 100 replicates. GenBank accession numbers are shown in parentheses. Scale bar = 0.50 substitutions per site.

Strain DGU8^T^ was compared with 96 draft genomes of *Paraburkholderia*, based on 3508 species and 81 single-copy bacterial core genes. *Paraburkholderia panacisoli* DCY113^T^ was identified as the closest clade, followed by *Paraburkholderia ribeironis* STM 7296^T^, *Paraburkholderia ultramafica* LMG 28614^T^, *Paraburkholderia ginsengisoli* FDAARGOS 1049^T^, and *P*. *fynbosensis* LMG 27177^T^ (Figure 3). The AAI phylogenetic tree strongly corroborated the UBCG2 analysis, with the same strains forming a distinct clade (Figure S1), thereby validating the selection of reference strains.

Comparison of RAST subsystem features revealed that strain DGU8^T^ exhibited superior counts in categories such as cell wall and capsule, motility and chemotaxis, phages, prophages, transposable elements, plasmids, and potassium metabolism. Detailed subsystem compositions are provided in Table S1. The antiSMASH analysis of strain DGU8^T^ identified biosynthetic gene clusters for 3 terpenes, 1 hydrogen cyanide, 1 arylpolyene, 1 NRPS-like, 1 homoserine lactone, 1 terpene precursor, 1 redox cofactor, and 1 T1PKS (Table S2).

**Figure 4:**
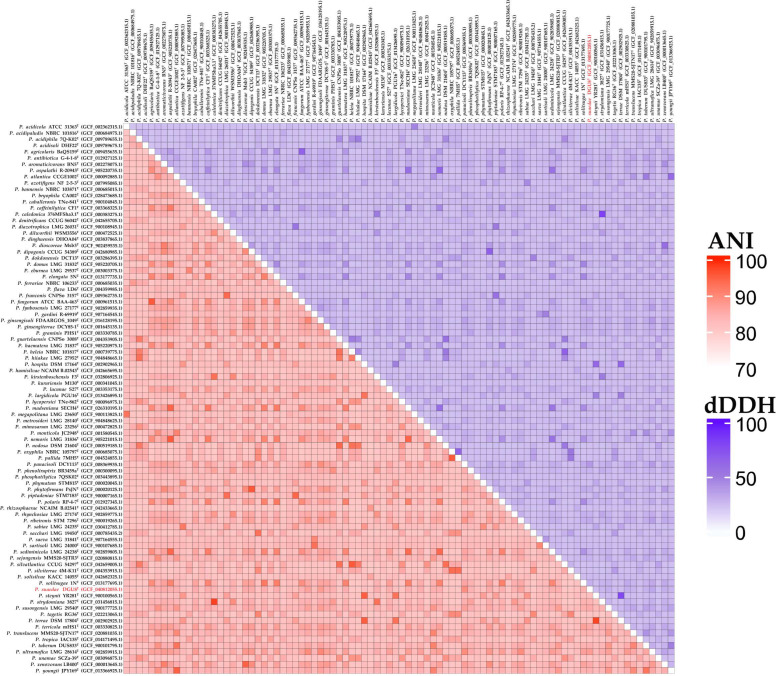
Heatmap matrix of average nucleotide identity (ANI) and digital DNA–DNA hybridization (dDDH) values for 88 *Paraburkholderia* species. Pairwise genome comparisons are displayed in a square matrix divided diagonally: the lower triangle presents ANI values in shades of red, while the upper triangle shows dDDH values in shades of purple. Color intensity reflects similarity levels, with darker shades indicating higher values.

The dDDH values between strain DGU8^T^ and reference strains mirrored the UBCG2 phylogenetic clade, with the top 5 matches belonging to the same clade. This indicates that the novel strain was genetically similar. The dDDH values were 38.0%, 32.7%, 31.6%, 31.1%, and 30.7% for *P*. *panacisoli* DCY113^T^, *P*. *ultramafica* LMG 28614^T^, *P*. *ginsengosoli* FDAARGOS 1049^T^, *P*. *fynbosensis* LMG 27177^T^, and *P*. *ribeironis* STM 7296^T^, respectively. The ANI values were 88.4%, 86.8%, 86.7%, 85.9%, and 85.5% for *P*. *panacisoli* DCY113^T^, *P*. *ginsengosoli* FDAARGOS 1049^T^, *P*. *ultramafica* LMG 28,614^T^, *P*. *fynbosensis* LMG 27,177^T^, and *P*. *ribeironis* STM 7296^T^, respectively. ANI and dDDH values for 88 *Paraburkholderia* species are visualized in the ANI/dDDH heatmap (Figure 4). A comprehensive comparison of ANI and dDDH values between the novel strain DGU8^T^ and 88 *Paraburkholderia* species is presented in the attached Supplementary Excel S2.

**Figure 5:**
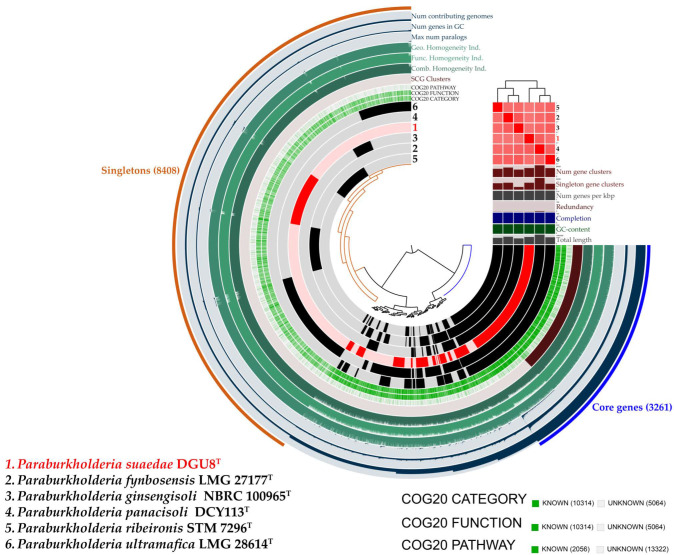
Pan-genome analysis of five reference strains and the novel strain *Paraburkholderia suaedae* DGU8^T^. Genomes are clustered based on the presence/absence of 15,378 gene clusters, illustrating shared and unique genomic content across strains.

The Anvi’o workflow was employed to validate the core genome and pan-genome structures of strain DGU8^T^ and five reference strains. The resulting pan-genome comprised 15,378 gene clusters, including 3261 core genes and 8408 singletons (genes unique to individual genomes). The outer edge of the pan-genome visualization illustrates the number of genomes containing specific gene clusters, the number of genes within those clusters, and the maximum number of paralogs (navy color). It also includes data on geometric, functional, and combined homogeneity indices (green), as well as the distribution of single-copy gene clusters (brown). Layers below the ANI heatmap represent, from bottom to top: total genome length, GC content, genome completeness, redundancy, gene density (genes per kbp), number of singleton gene clusters, and total gene cluster count (Figure 5).

**Figure 6:**
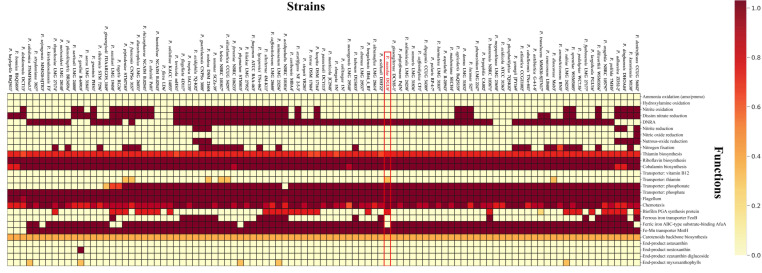
KEGG decoder heatmap based on KEGG annotations for strain DGU8^T^ and selected *Paraburkholeria* species. The heatmap illustrates the completeness of curated metabolic pathways, based on the presence/absence of genes, with an exclusive focus on functions related to plant growth promotion.

KEGG Decoder analysis revealed that strain DGU8^T^ exhibited high predicted values for genes involved in thiamine biosynthesis, thiamine transport, and biofilm PGA synthesis (Figure 6). Thiamin (vitamin B1) functions as a cofactor for enzymes involved in the biosynthesis of IAA, a key plant growth regulator that also contributes to defense responses and overall plant development [68]. The presence of thiamin transporter genes suggests that strain DGU8^T^ may interact with plants not only through thiamin biosynthesis but also via direct transfer pathways within the rhizosphere. Furthermore, genes associated with PGA-based biofilm synthesis provide a genetic basis for the potential to form biofilms, which could enhance adhesion and survival on root surfaces, thereby contributing to colonization and robust plant–microbe interactions [69,70]. Detailed proportions of EggNOG and KEGG annotations are presented in Figure S2. Based on the eggNOG functional annotation, genes associated with plant growth-promoting activities such as phosphate solubilization, pyrroloquinoline quinone (PQQ) production, nitrogen fixation, IAA production, and siderophore production were identified and are provided in Table 1.

### 3.4. Morphology and Chemotaxonomic Characteristics

Strain DGU8^T^ grew on both R2A and NA plates, with optimal growth observed on R2A agar. Strain DGU8^T^ was observed as circular, convex, and ivory-colored on R2A agar. It was capable of growing within a temperature range of 15–37 °C, with an optimum at 30 °C. The strain tolerated NaCl concentrations in the range of 0–1.0% (*w*/*v*), with optimal growth at 0%. However, no growth was observed at or above 2.0% NaCl. Furthermore, it grew within a pH range of 4.0–11.0, but no growth was observed below pH 3.0 or above pH 12.0.

**Figure 7:**
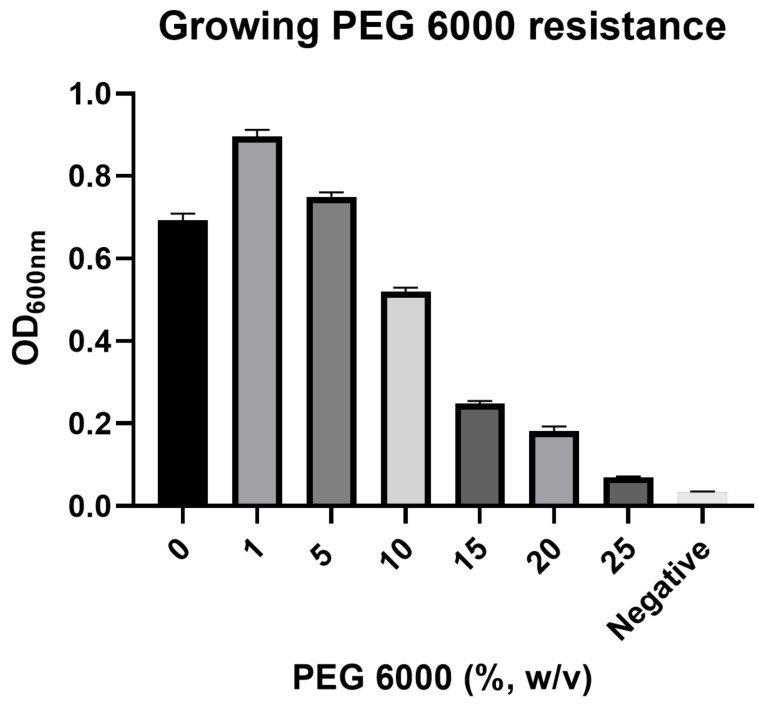
Drought resistance of *Paraburkholderia suaedae* DGU8^T^ against PEG-induced osmotic stress. The strain was cultured at 30 °C for 3 days in R2A broth supplemented with various concentrations of PEG 6000 (0–25%, *w*/*v*) to simulate drought conditions. Bacterial growth was determined by measuring the optical density at 600 nm (OD_600_). Bars represent the mean values of triplicate experiments, and error bars indicate the standard deviation.

The drought resistance of strain DGU8^T^ was evaluated under osmotic stress conditions simulated by PEG 6000. As shown in the results, the strain exhibited a clear tolerance to the tested stress levels. While the highest growth (OD_600_ ≈ 1.0) was observed in the control medium without PEG 6000, the strain maintained robust growth in the presence of 5% and 10% PEG 6000. Although growth was gradually inhibited in a dose-dependent manner, the strain still showed significant growth (OD_600_ ≈ 0.4) even at the highest concentration of 20% PEG 6000, confirming its potential to withstand severe drought stress (Figure 7).

The API 20 NE test indicated that strain DGU8^T^ was positive for nitrate reduction (NO_3_^−^ to NO_2_^−^), esculin hydrolysis (ESC), *β*-galactosidase activity (PNPG), and maltose assimilation (MAL), demonstrating its ability to reduce nitrates, hydrolyze esculin, and utilize both PNPG and maltose as carbon sources. Weak growth was observed under anaerobic conditions. Enzymatic hydrolysis activity was weakly positive only for carboxymethyl-cellulose; all other reactions were negative. The physiological and biochemical characteristics of strain DGU8^T^ compared to the reference *Paraburholderia* strains are detailed in Table 2.

TEM revealed that strain DGU8^T^ was rod-shaped and possessed a single flagellum (Figure S3). Catalase activity was confirmed by the generation of oxygen bubbles, while oxidase activity was negative.

The major fatty acid of strain DGU8^T^ was C_16:0_ (26.7%). Comparative fatty acid profiles with reference strains are detailed in Table S3. The predominant ubiquinone was Q-8.

### 3.5. Functional Traits Related to Plant Growth Promotion

**Figure 8:**
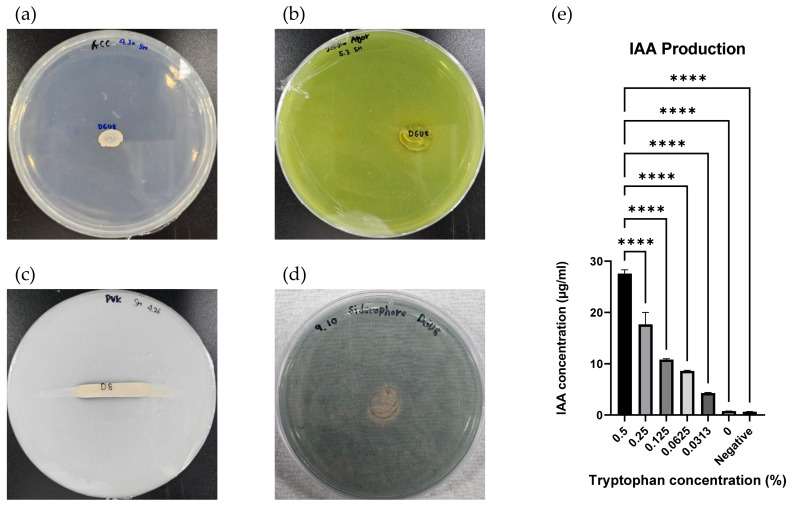
Functional evaluation of plant growth-promoting traits in strain *Paraburkholderia suaedae* DGU8^T^. The strain exhibited (**a**) 1-aminocyclopropane-1-carboxylic acid (ACC) deaminase activity, (**b**) nitrogen fixation, (**c**) phosphate solubilization, (**d**) siderophore production, and (**e**) indole-3-acetic acid (IAA) production (measured at 530 nm after 30 min incubation with Salkowski’s reagent), indicating its potential role in enhancing plant growth under various environmental conditions. All statistical analyses were performed using GraphPad Prism software version 11.0 for Windows (GraphPad Software, Inc., San Diego, CA, USA). According to Duncan’s test, significant differences between treatment group was: **** *p* < 0.0001.

Strain DGU8^T^ was able to grow on DF agar plates supplemented with ACC as the sole carbon source, demonstrating functional ACC deaminase activity (Figure 8a). It also grew and formed yellow halos on Jensen’s medium, indicating nitrogen fixation capability, which contributes to increased soil nitrogen availability and alleviation of salt stress in plants (Figure 8b).

Phosphate solubilization a trait that enhances soil fertility and supports crop development was evidenced by the formation of a narrow transparent zone, indicating a weak positive reaction (Figure 8c). Siderophore production was confirmed by the appearance of a transparent orange zone, indicating the strain’s ability to promote iron uptake in plants through siderophore secretion (Figure 8d).

Auxins are key plant hormones involved in growth and development, with IAA being one of the most extensively studied. IAA is produced via L-tryptophan metabolism [71]. Strain DGU8^T^ demonstrated IAA production in the presence of L-tryptophan, yielding 27.58 ± 0.63 μg/mL when 0.5% (*w*/*v*) L-tryptophan was added to the culture medium (Figure 8e), indicating its potential to promote plant growth.

In summary, strain DGU8^T^ exhibits multiple plant growth-promoting traits, including ACC deaminase activity, nitrogen fixation, phosphate solubilization, siderophore production, and IAA biosynthesis. These functional characteristics highlight its potential to enhance soil fertility, crop productivity, and overall plant health.

## 4. Discussion

In this study, we aimed to characterize a novel bacterium from the unique environment of a halophyte rhizosphere and to investigate its potential as a plant growth-promoting agent under abiotic stress. Our first objective was successfully met through a polyphasic taxonomic approach. The comprehensive genomic analysis, integrating various methods, provided conclusive evidence that strain DGU8^T^ represents a novel species within the *Paraburkholderia* genus. The UBCG2 phylogenetic tree clearly placed DGU8T in a distinct clade, and this taxonomic position was unequivocally supported by genome-wide metrics. The AAI, ANI, and dDDH values between DGU8^T^ and its closest relatives, such as *P*. *panacisoli* and *P*. *fynbosensis*, were all well below the established thresholds for species delineation (95–96% for ANI and 70% for dDDH) [53,54]. The visualization of genomic architecture with Anvi’o further substantiated these findings, revealing a unique genomic profile for our isolate. It is the first strain of the genus *Paraburkholderia* known to date to be isolated from a halophyte.

In line with our second objective, we explored the strain’s functional potential using genome annotation tools. The EggNOG and KEGG analyses revealed a rich repertoire of genes associated with plant growth promotion. Complete pathways for synthesizing key PGP compounds like IAA were identified, along with genes involved in phosphate solubilization and nitrogen fixation, whose effects were validated through In Vitro experiments [38,72,73]. The presence of these genes provided a strong genetic basis for the strain’s potential to enhance plant nutrition and development. Furthermore, the genome contained genes for thiamin biosynthesis and transport, which are known to play a vital role in plant health, and genes for PGA-based biofilm synthesis, suggesting a potential for root colonization.

A key theme of our study, introduced in the introduction, was the potential of microbial inoculants to enhance drought tolerance in plants. Our initial genomic findings were strongly supported by new experimental evidence. The in vitro PGP assays confirmed the functions predicted by the genome analysis, demonstrating that strain DGU8^T^ can produce significant amounts of IAA and solubilize phosphate. When we tested the strain’s resilience against drought-induced osmotic stress using PEG 6000, DGU8^T^ exhibited remarkable tolerance, maintaining substantial growth even at high concentrations that simulate severe drought conditions [74,75].

This result provides the direct experimental evidence linking this novel species to drought resistance, a critical trait for a bioinoculant intended for use in arid or water-limited agricultural systems. This finding addresses the question of whether strains isolated from non-crop plants, especially those from stressful environments like tidal flats, could be useful for sustainable agricultural production.

In conclusion, the isolation and characterization of *P. suaedae* DGU8^T^ contribute to our understanding of the microbial diversity in halophyte rhizospheres. By integrating robust genomic classification with functional genomics and targeted physiological assays, we have not only described a novel species but also provided strong evidence for its potential application in sustainable agriculture as a PGP agent with significant drought tolerance. The discovery of *P. suaedae* expands our understanding of the genus *Paraburkholderia*, particularly its ecological role in supporting plant development. Genomic insights confirmed the presence of key genes linked to nutrient mobilization and stress alleviation, suggesting potential applications in enhancing soil fertility and sustainable agriculture. Although this study was limited to in vitro assays, future research will include pot experiments to validate the strain’s plant growth-promoting efficacy under greenhouse and field conditions.

### Description of Paraburkholderia suaedae sp. nov.

*Paraburkholderia suaedae* sp. nov. (su.ae’dae. N.L. gen. n. *suaedae*, of *Suaeda*, the plant from which the strain was isolated).

Cells are Gram-negative, facultatively anaerobic, motile, and rod-shaped, and possess a single polar flagellum. Cell dimensions range from 2.10 to 2.42 μm in length and from 0.77 to 0.81 μm in width. Colonies are circular, convex, and ivory-colored. Growth occurs on R2A and NA media but not on MA, TSA, or LB. The strain grows at temperatures between 15 and 37 °C (optimum: 30 °C), pH 4.0–11.0 (optimum: pH 7.0), and NaCl concentrations of 0–1.0% (*w*/*v*; optimum: 0%). Hydrolysis reactions were observed only for CM-cellulose and catalase. In API 20 NE tests, the strain showed positive reactions for nitrate reduction, esculin hydrolysis, *β*-galactosidase activity, and maltose assimilation. The major fatty acid is C_16:0_, and the predominant ubiquinone is Q-8. The G + C content of the genomic DNA is 62.5%. The type strain is DGU8^T^ (= KACC 23737^T^ = TBRC 19126^T^), isolated from the root of *Suaeda japonica* on Seongmodo Island, Republic of Korea. The GenBank/EMBL/DDBJ/PIR accession numbers for the 16S rRNA gene and whole-genome sequences are PP702909 and JBFOAH010000000, respectively.

## Figures and Tables

**Table 1:** Genes associated with plant growth-promoting traits identified in the draft genome of strain *Paraburkholderia suaedae* DGU8^T^. Gene functions include phosphate solubilization, pyrroloquinoline quinone (PQQ) biosynthesis, nitrogen fixation, IAA production, and siderophore production. Annotation was performed using EggNOG.

Function	Start	End	Product	Gene
Phosphate solubilization	203,877	205,151	Phosphonoacetate hydrolase	*phn*A
	268,010	268,771	Phosphate-import ATP-binding protein *Phn*C	*phn*C_1
	281,941	282,801	Phosphate-import ATP-binding protein *Phn*C	*phn*C_2
	267,015	267,914	Putative ABC transporter phosphonate/phosphite binding protein *Phn*D2	*phn*D2
	268,768	269,568	Phosphate-import permease protein *Phn*E	*phn*E_1
	280,104	280,928	Phosphate-import permease protein *Phn*E	*phn*E_2
	277,734	278,492	putative transcriptional regulator *Phn*F	*phn*F
	277,076	277,558	Alpha-D-ribose 1-methyl phosphonate 5-triphosphate synthase subunit *Phn*G	*phn*G
	276,426	277,076	Alpha-D-ribose 1-methyl phosphonate 5-triphosphate synthase subunit *Phn*H	*phn*H
	275,287	276,426	Alpha-D-ribose 1-methyl phosphonate 5-triphosphate synthase subunit *Phn*I	*phn*I
	274,382	275,290	Alpha-D-ribose 1-methyl phosphonate 5-phosphate C-P lyase	*phn*J
	273,615	274,385	Putative phosphonates utilization ATP-binding protein *Phn*K	phnK
	272,839	273,606	Alpha-D-ribose 1-methyl phosphonate 5-triphosphate synthase subunit *Phn*L	*phn*L
	265,661	266,794	Alpha-D-ribose 1-methyl phosphonate 5-triphosphate diphosphatase	*phn*M_1
	271,672	272,805	Alpha-D-ribose 1-methyl phosphonate 5-triphosphate diphosphatase	*phn*M_2
	67,312	68,208	Alpha-D-ribose 1-methyl phosphonate 5-triphosphate diphosphatase	*phn*M_3
	279,238	279,795	Ribose 1,5-bisphosphate phosphokinase *Phn*N	*phn*N
	198,905	199,996	Putative 2-aminoethyl phosphonate-binding periplasmic protein	*phn*S
	200,094	201,194	Putative 2-aminoethyl phosphonate import ATP-binding protein *Phn*T	*phn*T
	202,112	202,975	Putative 2-aminoethyl phosphonate transport system permease protein *Phn*V	*phn*V
	18,652	18,798	Phosphonoacetaldehyde dehydrogenase	*phn*Y_1
	205,148	206,599	Phosphonoacetaldehyde dehydrogenase	*phn*Y_2
	206,604	207,164	2-Amino-1-hydroxymethyl phosphonate dioxygenase (glycine-forming)	*phn*Z
	332,262	332,963	Phosphate regulon transcriptional regulatory protein *Pho*B	*pho*B_1
	125,536	126,231	Phosphate regulon transcriptional regulatory protein *Pho*B	*pho*B_2
	124,094	124,777	Virulence transcriptional regulatory protein *Pho*P	*pho*P
	333,075	334,388	Phosphate regulon sensor protein *Pho*R	*pho*R
	331,531	332,235	Phosphate-specific transport system accessory protein *Pho*U	*pho*U_1
	34,391	35,098	Phosphate-specific transport system accessory protein *Pho*U	*phoU*_2
	45,975	46,682	Phosphate-specific transport system accessory protein *Pho*U	*pho*U_3
	334,488	336,551	Polyphosphate kinase	*ppk*
	336,733	338,367	Exopolyphosphatase	*ppx*
	329,732	330,628	Phosphate transport system permease protein *Pst*A	*pst*A
	330,646	331,503	Phosphate import ATP-binding protein *Pst*B	*pst*B
	328,737	329,735	Phosphate transport system permease protein *Pst*C	*pst*C
	327534	328565	Phosphate-binding protein *Pst*S	*pst*S
	180,222	181,160	Phosphate acetyltransferase	*pta*
	196,720	198,183	Glucose-6-phosphate 1-dehydrogenase	*zwf*_1
Pyrroloquinoline quinone (PQQ) production	207,713	208,651	Coenzyme PQQ synthesis protein B	*pqq*B
	208,714	209,481	Pyrroloquinoline-quinone synthase	*pqq*C
	209,478	209,768	PqqA binding protein	*pqq*D
	209,773	210,993	PqqA peptide cyclase	*pqq*E
Nitrogen fixation	123,632	124,045	Iron-sulfur cluster assembly scaffold protein *Isc*U	*isc*U
	140,604	141,059	Zinc-dependent sulfurtransferase *Suf*U	*suf*U
	92,432	94,072	Nitrogen fixation protein *Vnf*A	*vnf*A
Production of IAA	5454	7304	Tryptophan 2-monooxygenase	*iaa*M
	154,601	155,413	Tryptophan synthase alpha chain	*trp*A
	152,447	153,640	Tryptophan synthase beta chain	*trp*B
	14,333	15,118	Indole-3-glycerol phosphate synthase	*trp*C
	13,271	14,302	Anthranilate phosphoribosyltransferase	*trp*D
	11,120	12,613	Anthranilate synthase component 1	*trp*E
	151,687	152,421	N-(5′-phosphoribosyl)anthranilate isomerase	*trp*F
	12,627	13,253	Anthranilate synthase component 2	*trp*G
	5983	6861	HTH-type transcriptional regulator *Trp*I	*trp*I
	91,037	91,969	Tryptophan 2,3-dioxygenase	*kyn*A
Production of siderophore	46,942	48,519	4-Cresol dehydrogenase [hydroxylating] flavoprotein subunit	*pch*F
	5918	7165	Enterobactin exporter *Ent*S	*ent*S_1
	161,718	163,079	Enterobactin exporter *Ent*S	*ent*S_2
	137,907	139,169	Enterobactin exporter *Ent*S	*ent*S_3
	105,651	108,170	Acyl-homoserine lactone acylase *Pvd*Q	*pvd*Q

**Table 2:** Physiological and biochemical characteristics of strain *Paraburkholderia suaedae* DGU8^T^ compared with reference *Paraburkholderia* strains.

Characteristic	Strain					
	1	2	3	4	5	6
Colony color	Ivory	White	Cream	Yellow	White	White
Optimum growth temperature (°C)	30	25	30	30	30	30
Optimum growth pH	7	8	7	7	6	6
Catalase activity	+	+	+	+	+	+
Oxidase activity	–	+	–	+	–	+
Hydrolysis of:						
Casein	–	–	+	+	–	–
Chitin	–	–	–	–	–	–
Tween 20	–	–	–	–	+	–
Carboxymethyl cellulose	+	–	+	–	–	+
Assimilation of (API 20 NE):						
D-maltose	+	–	–	–	–	–
4-Nitrophenyl-*β*-d-galactopyranoside	+	+	+	–	+	+
Aesculin	+	+	+	+	–	+

Strains: 1, *P*. *suaedae* DGU8^T^; 2, *P. fynbosensis* LMG 27177^T^; 3, *P. ginsengisoli* NBRC 100965^T^; 4, *P. panacisoli* DCY113^T^; 5, *P. ribeironis* STM 7296^T^; 6, *P. ultramafica* LMG 28614^T^. +, positive; −, negative.

## Data Availability

The genomic data for strain DGU8^T^, comprising the Whole Genome Shotgun sequence obtained through Sanger sequencing and the 16S rRNA gene sequence, have been submitted to the DDBJ/ENA/GenBank databases. The accession numbers are JBFOAH010000000 for the whole genome and PP702909 for the 16S rRNA gene.

## Supplementary Materials

Supporting information related to this study is available at https://www.mdpi.com/article/10.3390/microorganisms13112498/s1. Figure S1: Phylogenetic tree based on average amino acid identity (AAI), constructed using strains analyzed in the UBCG2 refinement pipeline; Figure S2: Functional category distribution derived from the draft genome of strain DGU8^T^, annotated using the EggNOG and KEGG databases; Figure S3: Transmission electron microscopy image of strain DGU8^T^ cultured on R2A agar at 30 °C for 4 days, visualized using a JEM-1010 microscope (JEOL, Tokyo, Japan); Table S1: Subsystem characteristics of strain *Paraburkholderia suaedae* DGU8^T^ and five reference strains evaluated using the RAST annotation server; Table S2: Secondary metabolite gene cluster profiles of *Paraburkholderia suaedae* DGU8^T^; Table S3: Cellular fatty acids composition of novel strain DGU8^T^ and their respective closest reference strains; Excel S1: Genome differences among 97 species of *Paraburkholderia*.; Excel S2: ANI/dDDH values for 88 species of *Paraburkholdeira*.

## Abbreviations

The following abbreviations are used in this manuscript:

KEGG	Kyoto Encyclopedia of Genes and Genomes
ANI	average nucleotide identity
dDDH	digital DNA-DNA hybridization
ACC	1-aminocyclopropane-1-carboxylic acid
CAS agar	universal chrome azurol S agar
