# Supplementary material for: Paraburkholderia suaedae sp. nov., a Potential Plant Growth-Promoting Bacterium Isolated from the Halophyte Suaeda japonica
Source: Microorganisms. 2025 Oct 30;13(11):2498. doi: 10.3390/microorganisms13112498 (PMC12654207; doi:10.3390/microorganisms13112498)
Supplement: Supplementary file 1 [file microorganisms-13-02498-s001.zip › 3._Supplementary_Materials.v2.pdf]

microorganisms

Supplementary Materials

Paraburkholderia suaedae sp. nov., a Potential Plant Growth-Promoting Bacterium Isolated from the Halophyte *Suaeda japonica*

Sunho Park, Hyunji Lee, Subin Yook, Chunghwan Baek, Jisu Kim, Seunghui Kwak, Taeho Na and Taegun Seo *

Department of Life Science, Dongguk University-Seoul, Goyang 10326, Republic of Korea; eksvnd97@dgu.ac.kr (S.P.); guswl4851@dgu.ac.kr (H.L.); qw745043@gmail.com (S.Y.); qazx12300@naver.com (C.B.); didoo0v0@gmail.com (J.K.); sholly0@naver.com (S.K.); thth0417@dgu.ac.kr (T.N.)

* Correspondence: tseo@dongguk.edu; Tel.: +82-31-961-5135

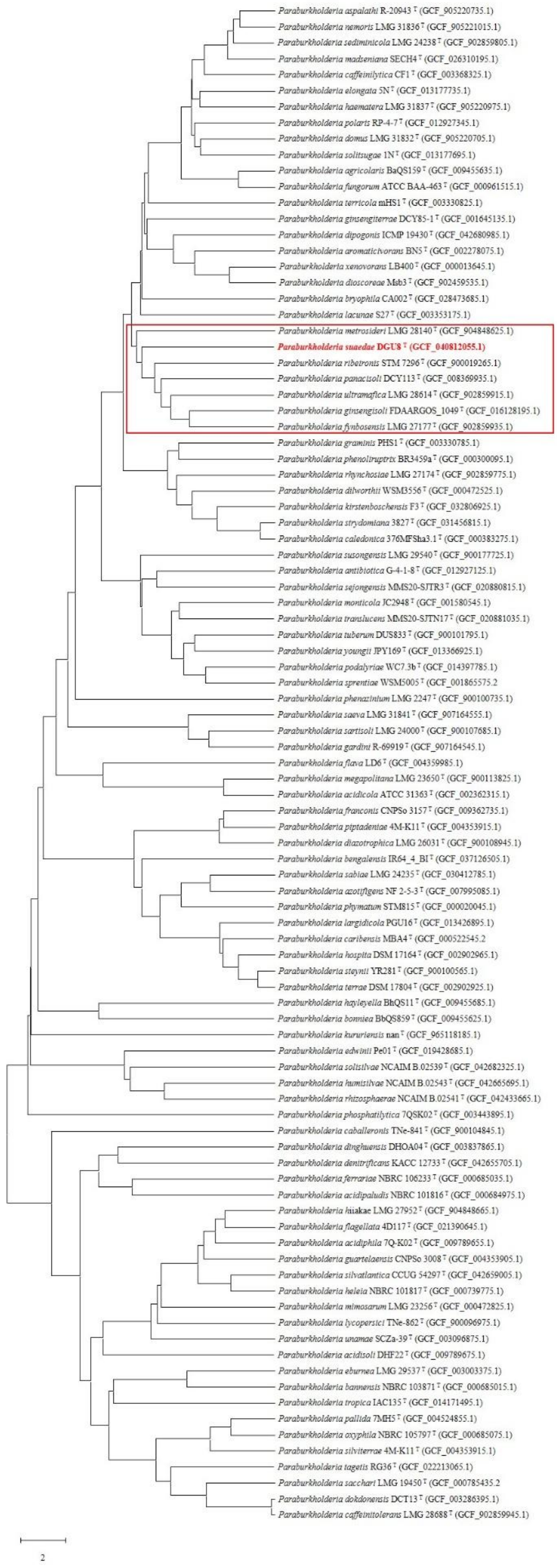

Figure. S1. Phylogenetic tree based on average amino acid identity (AAI), constructed using strains analyzed in the UBCG2 refinement pipeline.

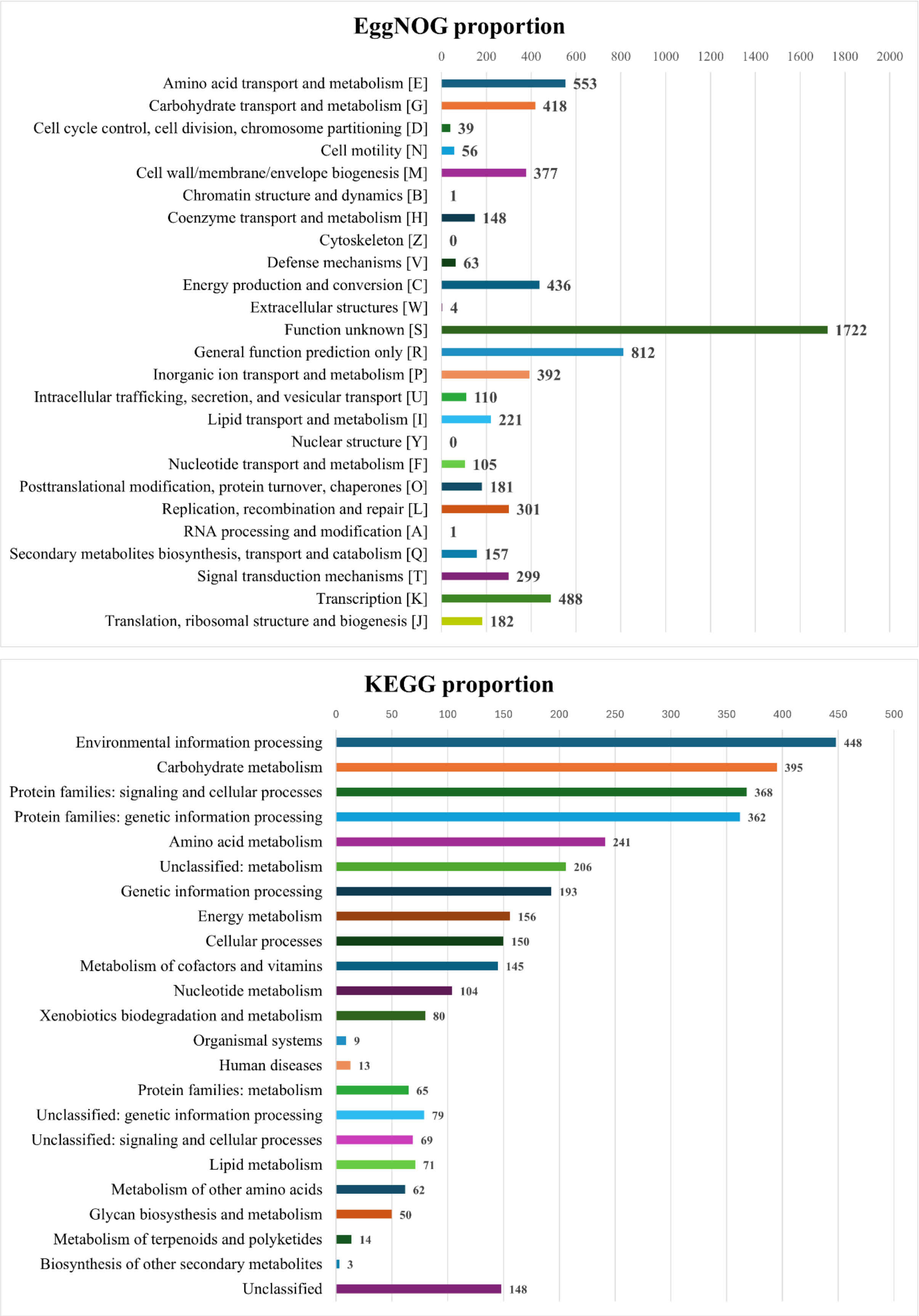

Figure. S2. Functional category distribution derived from the draft genome of strain DGU8^T, annotated using the EggNOG and KEGG databases.

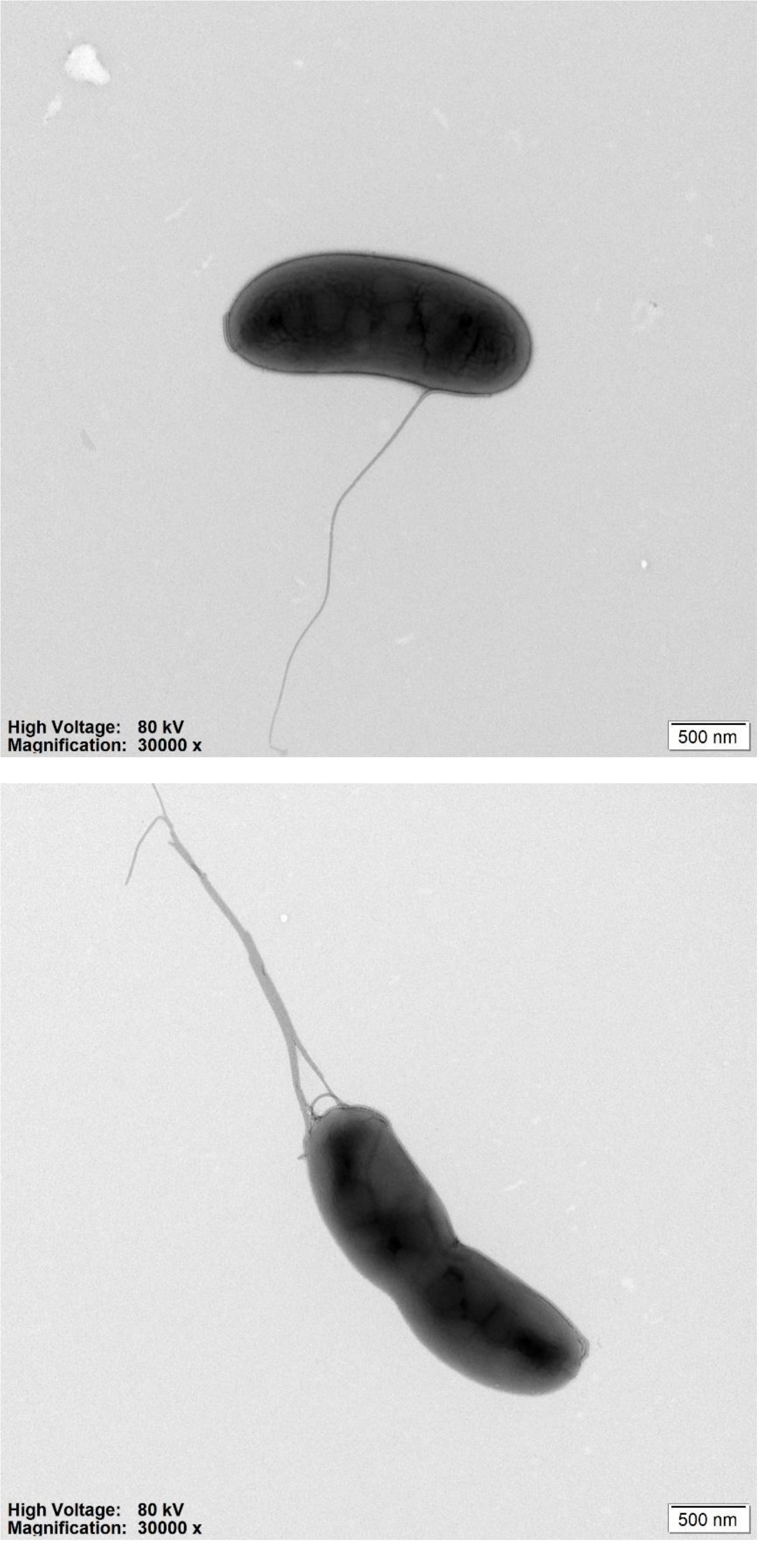

Figure. S3. Transmission electron microscopy image of strain DGU8^T cultured on R2A agar at 30°C for 4 days, visualized using a JEM-1010 microscope (JEOL).

Subsystem category distribution	Strain					
	1	2	3	4	5	6
Amino acids and derivatives	537	516	381	605	419	519
Carbohydrates	432	461	364	602	420	406
Cell division and cell cycle	0	0	0	0	0	0
Cell wall and capsule	53	41	47	42	35	51
Cofactors, vitamins, prosthetic groups, and pigments	225	216	200	269	214	212
DNA metabolism	102	99	83	113	88	111
Dormancy and sporulation	1	1	1	1	1	1
Fatty acids, lipids, and isoprenoids	130	109	95	173	102	110
Iron acquisition and metabolism	0	0	3	4	3	3
Membrane transport	48	59	53	57	71	54
Metabolism of aromatic compounds	126	115	100	228	119	126
Miscellaneous	43	54	50	97	40	60
Motility and chemotaxis	23	19	21	19	20	19
Nitrogen metabolism	16	12	12	16	32	13
Nucleosides and nucleotides	0	0	0	0	0	0
Phages, prophages, transposable elements, and plasmids	105	105	94	105	97	98
Phosphorus metabolism	0	2	2	0	0	2
Photosynthesis	30	42	44	56	30	42
Potassium metabolism	0	0	0	0	0	0
Protein metabolism	22	13	11	18	11	18
Regulation and cell signaling	212	214	226	216	216	217
Respiration	27	26	32	41	21	31
RNA metabolism	155	147	116	175	149	193
Secondary metabolism	54	52	49	77	60	56
Stress response	6	7	7	35	6	7
Sulfur metabolism	108	107	84	129	112	103
Virulence, disease, and defense	10	29	25	52	11	13

Table S1. Subsystem characteristics of strain *Paraburkholderia suaedae* DGU8^T and five reference strains evaluated using the RAST annotation server.

Strains: 1, *P. suaedae* DGU8^T; 2, *P. fynbosensis* LMG 27177^T; 3, *P. ginsengisoli* NBRC 100965^T; 4, *P. panacisoli* DCY113^T; 5, *P. ribeironis* STM 7296^T; 6, *P. ultramafica* LMG 28614^T.

Region	Type	From	To	Most similar known cluster	Similarity confidence
1.1	Terpene	578,853	599,677	–	–
1.2	Hydrogen–cyanide	734,338	748,415	–	–
2.1	Arylpolyene	84,599	129,463	APE Vf	Low
4.1	NRPS–like	94,766	137,831	O-antigen	Low
5.1	Terpene	43,349	76,167	–	–
5.2	Terpene	186,867	207,913	–	–
5.3	Hserlactone	226,051	246,644	–	–
8.1	Terpene–precursor	204,867	225,748	–	–
11.1	Redox–cofactor	198,714	213,773	–	–
15.1	T1PKS	88,374	134,718	–	–

Table S2. Secondary metabolite gene cluster profiles of *Paraburkholderia suaedae* DGU8^T.
NRPS-like, non-ribosomal peptide synthetase-like fragment; T1PKS, Type I polyketide synthase.

Fatty acid	Strain					
	1	2	3	4	5	6
Saturated						
$C_{11:0}$	2.1	3.1	1.5	1.1	TR	TR
$C_{12:0}$	7.6	5.6	4.8	3.6	–	–
$C_{14:0}$	2.9	4.4	5.5	3.7	10.5	3.1
$C_{16:0}$	26.7	17.9	16.2	30.1	22.4	8.8
$C_{18:0}$	9.7	TR	TR	4.5	TR	TR
Unsaturated						
$C_{19:0}$ cyclo *ω*8*c*	6.8	2.7	15.3	9.0	3.0	3.1
$C_{14:1}$ *ω*5*c*	1.4	1.1	3.2	2.6	TR	TR
Hydroxy						
$C_{11:0}$ 2OH	1.3	1.4	3.5	TR	2.3	TR
$C_{16:0}$ 2OH	3.7	1.5	5.1	TR	TR	1.5
$C_{16:0}$ 3OH	5.8	7.5	8.4	4.2	8.3	4.7
$C_{16:1}$ 2OH	1.6	2.1	2.3	TR	TR	1.4
Branched						
$C_{17:0}$ cyclo	6.1	12.4	22.1	13.6	15.2	10.0
$C_{11:0}$ anteiso	2.0	TR	TR	1.2	TR	1.8
$C_{13:0}$ anteiso	1.7	1.2	1.1	TR	TR	1.9
$C_{14:0}$ anteiso	1.2	TR	N/D	TR	1.0	1.6
$C_{15:0}$ anteiso	1.0	TR	TR	TR	1.2	1.1
$C_{18:1}$ *ω*8*c* 11-methyl	1.0	TR	N/D	N/D	TR	1.2
Summed features:						
**3***	1.3	13.0	2.3	5.6	9.9	3.4
**8***	3.8	25.5	4.0	14.9	15.8	33.1

Table S3. Cellular fatty acids composition of novel strain DGU8^T and their respective closest reference strains. Strains: 1, *P. suaedae* DGU8^T; 2, *P. fynbosensis* LMG 27177^T; 3, *P. ginsengisoli* NBRC 100965^T; 4, *P. panacisoli* DCY113^T; 5, *P. ribeironis* STM 7296^T; 6, *P. ultramafica* LMG 28614^T.

* Summed features represent fatty acids that cannot be reliably separated due to chromatographic limitations. The MIDI Sherlock Microbial Identification System (MIS) groups co-eluting or hybridized fatty acids as a single percentage of the total. Summed feature 3 includes $C_{16:1}$ *ω6c* and/or $C_{16:1}$ *ω7c* and summed feature 8 includes $C_{18:1}$ *ω7c* and/or $C_{18:1}$ *ω6c*. TR, trace amount (<1.0% of the total); –, not detected.
